# Breaking down the Dyslipidemia–Stroke Relationship in Southeast Asian American Subgroups: Advancing Toward Cardiovascular Health Equity

**DOI:** 10.1007/s40471-025-00376-4

**Published:** 2025-12-19

**Authors:** Joshua Vo, Youssef M. Roman

**Affiliations:** 1 Virginia Commonwealth University School of Medicine, Richmond, VA 23298, USA; 2 L.S. Skaggs College of Pharmacy, Idaho State University, 1311 E Central Dr, Meridian, ID 83642, USA

**Keywords:** Dyslipidemia, Stroke, Health disparities, Asian health, Data aggregation

## Abstract

**Purpose of Review:**

Southeast Asian (SEA) American populations—including Filipino, Vietnamese, Cambodian, Laotian, Burmese, and Hmong communities—experience disproportionate burdens of dyslipidemia and cardiovascular disease in the United States (U.S.) population. Despite these disparities, SEA Americans remain underrepresented in cardiovascular research and epidemiology. This review synthesizes U.S.-based epidemiologic, clinical, and community-level evidence on dyslipidemia and stroke across SEA subgroups, emphasizing how aggregated data obscure subgroup-specific disparities.

**Recent Findings:**

Disaggregated data reveal that Filipino and Vietnamese adults—among the most studied SEA subgroups—exhibit high rates of elevated low-density lipoprotein cholesterol and hypertriglyceridemia, aligning with increased ischemic stroke prevalence, higher age-standardized cerebrovascular mortality, and greater years of potential life lost. Hemorrhagic stroke mortality is also comparatively high in these groups. In contrast, data for Cambodian, Laotian, Hmong, and Burmese Americans remain sparse, limiting risk characterization. Emerging evidence highlights that cardiometabolic disorders in SEA populations reflect heterogeneous and multifactorial influences, including genetic variability in statin metabolism, cultural health beliefs, psychosocial stressors, and systemic barriers to preventive care.

**Summary:**

Cardiovascular risk in SEA American populations is shaped by the interplay of biological and social determinants of health. Aggregating diverse Asian subgroups into a single racial category masks heterogeneity, widens knowledge gaps, and perpetuates inequities in care. Advancing cardiovascular health equity requires intentional inclusion of underrepresented SEA subgroups in clinical research, systematic data disaggregation, and culturally responsive approaches to lipid management and stroke prevention.

## Introduction

Cardiovascular diseases (CVD) are the leading cause of death nationally, with heart disease accounting for 26.2% of all deaths [1]. The rates of CVD, which include hypertension, congenital heart disease, heart failure, and stroke, are increasing. By 2030, approximately 40% of the United States (U.S.) population is predicted to develop some form of CVD [2]. Additionally, dyslipidemia affects more than 11% of adults aged 20 and older [3]. Previous literature has shown that dyslipidemia disproportionately affects distinct racial and ethnic subgroups. For example, according to the INTERHEART study, 61.5% of South Asians exhibited elevated lipoproteins compared to other groups at 48.3% [4].

Despite Asian Americans being one of the fastest growing populations in the U.S. and expected to become the largest immigrant group by 2065, there remain significant gaps in knowledge about their health [5]. Southeast Asian (SEA) Americans, specifically, are considered a high-risk demographic due to their significant burden of cardiovascular risk factors [6]. The SEA population consists of diverse subgroups—including, but not restricted to, Vietnamese, Filipino, Cambodian, Laotian, Burmese, and Hmong—yet health data specific to these communities is limited [7]. Historically, Asian populations have been grouped into a monolithic category that, as an aggregate, appeared healthier than non-Hispanic Whites (NHW) on most indicators. However, when disaggregating into subgroups, every Asian subgroup had at least one disparity that had been masked by aggregation [8].

As the prevalence of cardiometabolic disorders continues to rise, stroke has proven to be one of the most serious complications, accounting for approximately 17.5% of cardiovascular deaths in the U.S. population [9]. Among the key risk factors for stroke, dyslipidemia plays a critical role due to its contribution to atherosclerosis and impaired cerebral blood flow [10, 11]. For SEA American populations, who already face a heightened burden of CVD risk, examining the intersection between dyslipidemia and stroke for each subgroup remains a significant yet under-explored public health concern and a major knowledge gap [12].

Although SEA American subgroups face higher cardiovascular risk, they are still significantly underrepresented in research, resulting in limited evidence for developing tailored prevention and treatment strategies. This review compiles current research on dyslipidemia and stroke risk among SEA populations, focusing on the extent and quality of subgroup data disaggregation and its clinical implications. By identifying key knowledge gaps, we aim to guide future research designs and promote clinical frameworks that support subgroup-specific screening, risk assessment, and treatment—advancing beyond the common one-size-fits-all approach to Asian American cardiovascular care.

## Methods

This narrative review synthesized peer-reviewed literature published from 2000 through April 2025 using PubMed and Google Scholar. Eligible studies were written in English and focused on SEA Americans, Asian American immigrants, or U.S.-born Asians. Non-U.S. studies were excluded to ensure the findings reflected cardiovascular risk patterns relevant to Asian American populations. We also included select historical and sociocultural sources for contextual framing. Evidence was synthesized thematically across three domains: (1) the dyslipidemia-stroke relationship, (2) subgroup differences in dyslipidemia prevalence, and (3) subgroup differences in stroke risk.

## Results

### Dyslipidemia-Cerebrovascular Accident Pathogenesis

Cerebrovascular accidents (CVAs) arise from disrupted cerebral blood flow and are classified as ischemic (arterial obstruction) or hemorrhagic (vessel rupture) [13]. Dyslipidemia is a major risk factor, most strongly linked to ischemic stroke (IS)—the predominant subtype in the U.S. (82.7% in 2019) [14–16]. It reflects abnormal lipid metabolism: elevated total cholesterol (TC), low-density lipoprotein cholesterol (LDL-C), and triglycerides (TG), or reduced high-density lipoprotein cholesterol (HDL-C) [17]. High TC and LDL-C increase IS risk, HDL-C is generally protective, and higher TG—marking remnant lipoproteins—also elevates IS risk [15, 18–21].

Mechanistically, dyslipidemia promotes chronic inflammation, oxidative stress, endothelial dysfunction, and plaque formation, narrowing cerebral arteries, impairing perfusion, and increasing thrombosis [17, 22, 23]. Despite debate about hemorrhagic events, lowering LDL-C reduces overall stroke incidence and mortality. The 2018 American College of Cardiology/American Heart Association Cholesterol Guideline therefore recommends generally maintaining LDL-C ≤ 100 mg/dL, and randomized trial meta-analyses show LDL-C-lowering—particularly with statins—reduces Atherosclerotic Cardiovascular Disease (ASCVD) events and overall stroke mortality [17, 24–26].

The relationship with hemorrhagic stroke (HS) is less certain. Observational studies associate low TC and LDL-C with higher intracerebral hemorrhage risk, raising concern that very low cholesterol may weaken vascular integrity [27, 28]. Other evidence finds no relationship between hemorrhagic risk and the degree of LDL-C reduction. A modest statin-associated increase in HS has been reported without a dose-response to LDL-C lowering, suggesting that non-lipid mechanisms (e.g., off-target drug effects or small-vessel pathology) may contribute [27–31]. Overall, these signals for HS are inconsistent and small, relative to the established benefits of LDL-C reduction on total stroke and major vascular events.

### Dyslipidemia Prevalence by Population Subgroup

Dyslipidemia is prevalent among SEAs (Table 1), with Filipino and Vietnamese adults showing the highest risk compared with NHW and other Asian subgroups [5, 32, 33]. Among men, 73% of Filipinos and 71% of Vietnamese have LDL-C ≥ 130 mg/dL; in contrast to 55% in Chinese and 62% in NHW adults [33]. High TGs (≥ 150 mg/dL) occur in 60% of Filipino men and 56% of Vietnamese men [33]. Women show similar patterns: high LDL-C in 63% of Filipinas and 56% of Vietnamese women versus 52% of NHW women, with low HDL-C (≤ 50 mg/dL) and high TGs more frequent than in NHW and East/South Asian counterparts [33].

**Table 1:** Dyslipidemia by subgroup

		Sex	Lipid Parameters	Vietnamese	Filipino	Chinese	Aggregate Asian*	NHW
Frank et al. [33]	Age-Standardized Prevalence Rates (%) of Dyslipidemia (2008-2011)	Men	High LDL-C Ever	71.3%	73.1%	55.3%	ND	62.2%
			Low HDL-C ever	33.6%	37.1%	34.0%	24.5%*	35.7%
			High TG Ever	55.6%	60.3%	48.7%	ND	42.5%
		Women	High LDL-C Ever	56.1%	63.0%	45.8%	ND	52.6%
			Low HDL-C ever	37.2%	37.3%	31.8%	5.1%*	30.7%
			High TG Ever	39.0%	41.5%	29.9%	ND	27.6%

* Aggregated Asian estimates derived from Pu et al. [91]

*ND* No data reported for this subgroup, *NHW* Non-Hispanic White

Regional clinical datasets align with these trends: Vietnamese adults show a 52.5% prevalence of high LDL-C, and Filipinos have the greatest prevalence of high TGs at nearly 50% [34]. Overall, SEAs appear more vulnerable to a cluster of multiple lipid abnormalities than East Asian groups such as Chinese and Korean adults [33]. The most recent preliminary PANACHE 2025 findings remain consistent, with Filipinos reporting the highest rates of high TC at 33% and Chinese the lowest at 20% [35].

Other SEA subgroups—including Cambodian, Laotian, and Hmong populations—likely face disproportionate cardiometabolic risk as well but remain underrepresented in national datasets [5, 32]. In the National Health Interview Survey 2010–2018, SEAs collectively reported the highest hyperlipidemia prevalence at 36% among Asian ethnicities [12]. Even for better-studied groups such as Vietnamese Americans, limited disaggregated national data constrain broader epidemiologic insight [36].

### Stroke Risk by Population Subgroup

Among SEA Americans, stroke burden is not evenly distributed (Tables 2 and 3). Specifically, Filipino and Vietnamese populations show disproportionately high cerebrovascular disease by age-standardized mortality rate (ASMR), proportionate mortality ratio (PMR), and years of potential life lost (YPLL), while data for other SEA subgroups remain limited [12, 37–40].

**Table 2:** Overall stroke risk by subgroup

		Vietnamese	Filipino	Chinese	Aggregate Asian	NHW
Iyer et al. [37]	Total cerebrovascular disease deaths, %	9.0	8.8	8.1	8.0	5.5
	Mean age at death because of cerebrovascular disease (2003–2012) (yrs)	72.5	74.3	79.3	76.8	80.8
	YPLL (2003–2012)	17	16	12 to 13	14 to 15	11 to 13
	YPLL per 100,000 (2012, men)	261	352	~ 175	~ 225	143
	YPLL per 100,000 (2012, women)	~ 330	306	195	250	~ 190
Shah et al. [38]	ASMR from CVAs per 100,000 (2017, men)	47.1	44.4	32.7	37.1	37.4
	ASMR from CVAs per 100,000 (2017, women)	45.7	33.6	27.9	33.1	38.4

*ASMR *age-standardized mortality rate, *CVA *cerebrovascular accident, *YPLL *years of potential life lost

**Table 3:** Hemorrhagic stroke risk by subgroup

		Sex	ASMR per 100,000	PMR
Jose et al. [41]	Vietnamese	Women	13.7	3.4
		Men	15.2	2.9
	Filipino	Women	13.3	2.9
		Men	18.3	2.6
	Chinese	Women	10.1	2.3
		Men	13.1	2.2
	White	Women	9.3	1.1
		Men	11.3	0.9

*ASMR *age-standardized mortality rate, *PMR *proportionate mortality ratio

### Vietnamese

In 2017, Vietnamese men and women had the highest stroke ASMR among Asian subgroups at 47 and 46 per 100,000, respectively [38]. Unlike Chinese, Filipino, and Japanese groups, Vietnamese ASMR did not decline from 2003 to 2017, indicating a persistent disparity [38]. Stroke PMR was also highest at 1.8 in men and 1.7 in women versus aggregate Asian PMR of 1.6 and 1.4, respectively [38]. HS risk was particularly elevated: women HS ASMR 13.7 and PMR 3.4; men HS ASMR 15.2 and PMR 2.9, all exceeding NHW benchmarks of 9.3 and 1.1 in women and 11.3 and 0.9 in men [41]. Premature mortality is substantial, with mean YPLL of 17 years in both sexes, the lowest mean age at stroke death at 72.5 years, and persistently higher YPLL per 100,000 than aggregate Asian adults [37].

### Filipino

Filipinos had the highest age-adjusted prevalence of IS among Asian subgroups at 1.8% in a large outpatient cohort [39]. HS burden was also high, with ASMR 18.3 in men and 13.3 in women and PMR 2.6 and 2.9; intracranial hemorrhage risk was 2.3-fold higher than in NHW adults [39, 41]. Overall CVA ASMR declined from 2003 to 2017 by about 2.9% per year in men and 2.8% per year in women yet remained above aggregated Asian averages in 2017 at 44 in men and 34 in women; PMR also stayed elevated at 1.7 and 1.5, respectively [38]. Premature mortality remains pronounced, with a mean YPLL of 16 years and the highest YPLL per 100,000 across the study period in both sexes, declining from 404 to 352 in men and from 474 to 306 in women [37].

### Other Subgroups

While stark disparities are evident for Filipino and Vietnamese Americans, disaggregated stroke data are largely absent for Cambodian, Lao, Hmong, Burmese, and Thai populations. None of the cited studies provides subgroup-specific stroke estimates for these groups. Many reports aggregate SEAs or all Asian Americans, masking distinct risks. Consequently, only Filipino and Vietnamese populations are consistently analyzed, reflecting larger numbers and greater visibility in public datasets [36]. This lack of subgroup-specific data limits a full understanding of cerebrovascular disease burden across SEA communities.

## Discussion

Asian American subgroups do not share a uniform cardiovascular risk profile. Instead, there is marked heterogeneity in CVD prevalence, risk factors, and disease patterns across ethnic lines—particularly among Southeast, East, and South Asian populations [12]. Even within the SEA category, distinct subgroup differences emerge, shaped by a complex interplay of genetic predispositions, cultural practices, dietary habits, and socioeconomic factors that collectively influence cardiovascular health and drive health disparities in stroke and dyslipidemia [32, 42].

Among these factors, variation in dyslipidemia prevalence stands out as a key contributor to elevated CVA risk in several SEA American populations [32–34, 40, 41, 43]. Such disparities emphasize the necessity of disaggregating data within the Asian American population to more precisely identify and address subgroup-specific cardiovascular risks as well as the determinants of treatment response [44]. This approach is vital for informing targeted, culturally responsive clinical interventions [5].

While aggregate statistics portray Asian Americans as having lower overall rates of CVD risk factors and stroke, this broad categorization conceals the elevated burden of cerebrovascular disease in certain subgroups [45]. For example, Vietnamese and Filipino Americans experience disproportionately higher burdens of cerebrovascular disease compared to NHWs and the broader Asian American population [37, 38]. While YPLL per 100,000 declined across most racial and ethnic groups—including among Filipino and Vietnamese Americans—both subgroups consistently exhibited the highest levels throughout the study period, underscoring a persistent disparity in premature stroke-related mortality [37]. Moreover, YPLL alone fails to account for contributing factors such as comorbidities, healthcare access, or subgroup-specific biological variation, driven by genetic heterogeneity, limiting its explanatory power and reinforcing the need for more granular epidemiologic data. Together, these patterns point to deeper, underlying risk factors that likely drive the sustained disparities in stroke-related mortality observed in these groups. One such factor is elevated LDL-C, which remains more prevalent among Filipino and Vietnamese Americans compared to other East Asian subgroups and NHW [33, 34].

**Fig. 1:**
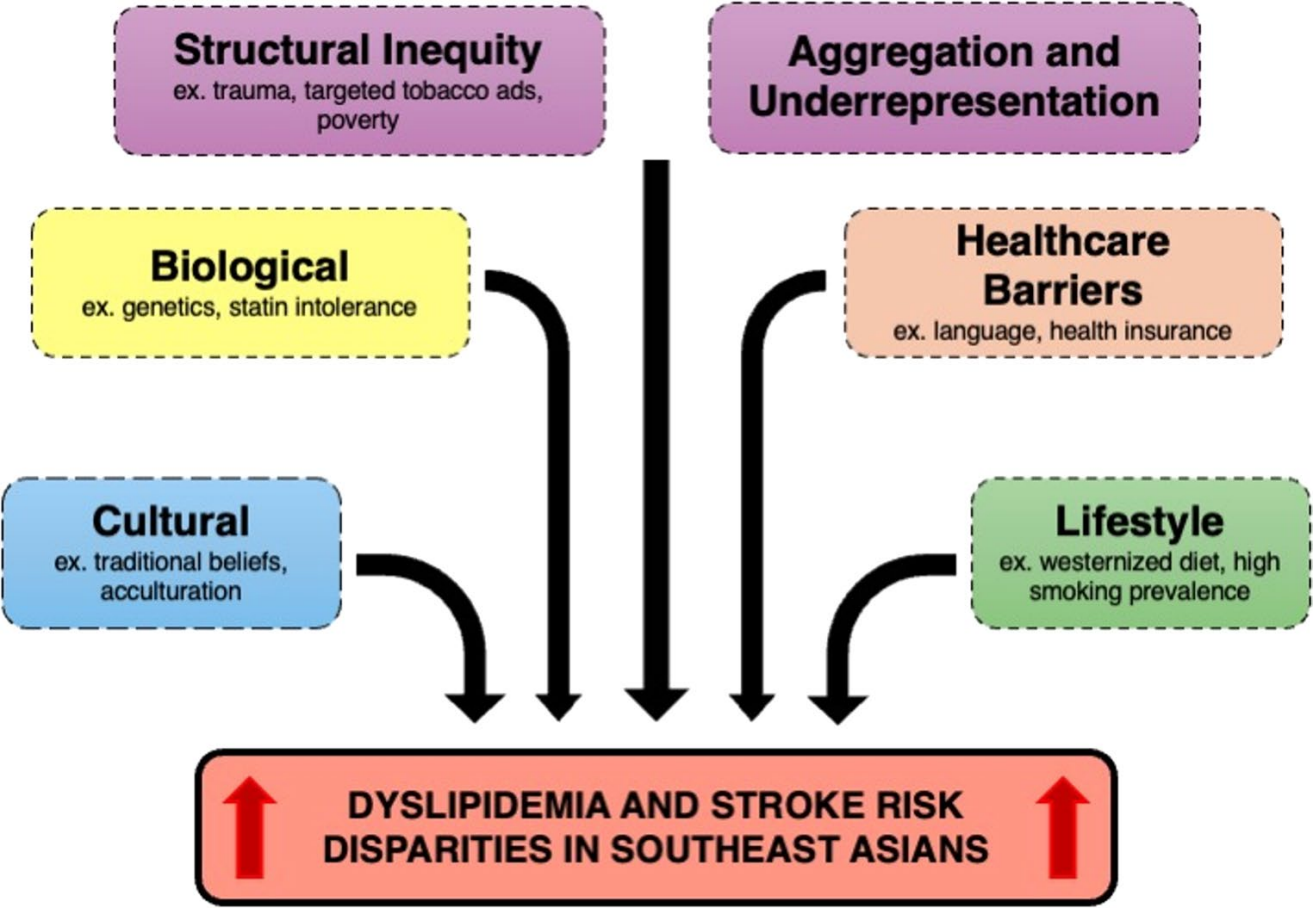
Conceptual framework for dyslipidemia and stroke risk disparities among Southeast Asian Americans. Factors across structural (e.g., trauma, targeted tobacco marketing, poverty), data/representation (aggregation and underrepresentation), healthcare access (e.g., language, insurance), lifestyle (e.g., diet, smoking), biological (e.g., genetic variation, statin intolerance), and cultural domains (e.g., beliefs, acculturation) interact and converge to influence risk and outcomes; arrows indicate convergence and correlation, not causality

The increased rates of dyslipidemia among these SEA subgroups may also be attributable to sociocultural factors, the interplay of genetic inheritance, and long-standing structural inequities and underrepresentation (Fig. 1). At the behavioral level, smoking illustrates this intersection: Vietnamese men have the highest prevalence of current smoking (29.5%) among Asian American groups [45]. Smoking, through its association with atherogenic lipid profiles, may increase risk not only for stroke but across the entire CVD spectrum, thereby compounding disparities in this population [46–48].

These patterns of elevated smoking rates cannot be viewed in isolation, but rather as outcomes shaped by socioeconomic marginalization and cultural influences. SEA American subgroups such as Vietnamese, Cambodian, Laotian, Hmong, and Burmese populations face disproportionately low average wages compared to the Asian American Pacific Islander and U.S. total average, lower household income, and higher rates of poverty [49–53]. These socioeconomic disparities place these populations at risk for targeted marketing by the tobacco industry [54]. Low-income and ethnic minority communities, including SEA populations, are more likely to be exposed to high tobacco outlet density, exterior advertisements, and flavored or discounted products, particularly in immigrant-dense urban neighborhoods [55–58]. Outside of the U.S. tobacco industry’s active targeting of this minority group, the increased prevalence of smoking can also be attributed to cultural beliefs—such as perceptions of smoking as a symbol of strength, peer dynamics, and limited health literacy regarding tobacco risks [36, 59].

Beyond tobacco use, dietary patterns shaped by acculturation, cultural norms, and economic access significantly contribute to dyslipidemia disparities among SEA Americans. The “negative acculturation effect” describes how longer residence in the U.S. is often associated with worsening health outcomes, particularly due to the adoption of Western dietary practices [60]. Many immigrants transition from traditional diets to Americanized patterns that are high in saturated fat, sugar, and sodium—factors strongly linked to elevated LDL-C and increased cardiovascular risk [61]. Across SEA immigrants, globalization-driven pre-acculturation familiarizes people with Western foods before migration, so after arrival, many adopt a bicultural diet that fuses traditional diets with accessible U.S. convenience foods [42]. This blend tends to be more energy-dense and sodium-rich while displacing nutrient-dense staples—patterns well-documented among Filipino immigrants and likely applicable to other SEA groups in similar food environments [62, 63]. Together, these shifts help explain in part elevated dyslipidemia risk and point to the need for subgroup-sensitive nutrition counseling.

These dynamics contribute to nutritional patterns that significantly elevate cardiometabolic disease risk, particularly when reinforced by reduced physical activity, psychosocial stress, and limited access to healthcare. Studies consistently show that longer U.S. residence is associated with increased fat intake, higher body mass index, and greater cardiometabolic burden—even after adjusting for age, gender, and education [60]. These risks may be even more pronounced in admixed households, where individuals must navigate competing dietary norms and cultural expectations. Such internal tensions can lead to inconsistent lifestyle choices, reduced social support, and heightened stress, all of which complicate cholesterol management and stroke prevention [32]. Thus, culturally responsive dietary counseling must go beyond food content to engage with the broader historical, psychological, and communal forces that shape eating behaviors and health outcomes in SEA communities.

Beyond the household level and building on the sociocultural contributors to elevated lipid levels, the refugee experience provides another layer of complexity in understanding cardiovascular risk. Though not all SEA Americans are refugees, approximately 60% of all people of Asian descent living in the U.S. were born outside of the country [64]. The CVD patterns identified in refugee and immigrant communities can provide valuable insight into broader subgroup-specific health vulnerabilities. Refugees from countries like Vietnam, Cambodia, and Burma face a unique combination of structural, cultural, and clinical risk factors, paralleling trends seen across the larger SEA American population. As highlighted in a recent integrative review by Bang et al., these populations suffer disproportionately from chronic conditions such as hypercholesterolemia, hypertension, and diabetes—each of which elevates the risk of cerebrovascular events [65]. Burmese American refugees, for instance, exhibited significantly higher LDL-C levels compared to a cohort of adults living in Burma [66]. Differences in lipid profiles between SEA refugees and populations in their home countries demonstrate that the elevated prevalence of dyslipidemia among SEA Americans is not shaped in isolation. Rather, it is influenced by a multitude of factors, including systemic barriers to healthcare such as inadequate insurance coverage, language barriers, and limited transportation access [65, 67–71]. These challenges—compounded by the psychosocial stress of resettlement, including trauma, poverty, and social isolation—exacerbate health disparities among SEA refugees (Fig. 1) [42, 65].

In addition to structural and social barriers, deep-seated cultural dissonance with the U.S. healthcare system plays a critical role in the underutilization of preventive and treatment services for conditions like high cholesterol. In exploring SEA health beliefs, Uba outlines how cultural attitudes toward suffering, illness etiology, and medical authority often lead individuals to delay or avoid seeking care [70]. For example, subgroups such as the Hmong may interpret physical, emotional, and psychological distress as a form of spiritual imbalance rather than a physiological issue, prompting reliance on traditional healers or ritual practices instead of Western medical intervention [70, 72, 73].

Communication breakdowns are also rooted in linguistic gaps and a limited understanding of Western medicine. Phillips et al. observed that, compared to other Asian subgroups, SEA immigrants were more likely to have limited English proficiency and cultural familiarity, which contributed to reduced comprehension during healthcare interaction [74]. While no study, to our knowledge, has directly examined the link between English proficiency and adherence to cholesterol-lowering medications in SEA subgroups, related research shows that non-English-speaking populations tend to have significantly higher LDL-cholesterol levels [75]. This is largely because they are less likely to be prescribed statins or other lipid-lowering therapies [75]. Exacerbating this issue, limited English proficiency among Asian Americans has been associated with increased odds of lacking a usual source of care, missing regular checkups, having unmet medical needs, and experiencing miscommunication in healthcare settings [76, 77]. Building on these language barriers, certain SEA subgroups, like the Hmong, are more prone to question the need for treatment of conditions that have not yet presented with visible symptoms [78]. Similarly, Filipino Americans have a reserved perspective on Western medicine drugs, primarily due to the pluralism of medicine and culturally informed beliefs about health [42, 79].

Subgroup-specific differences in pharmacologic response to dyslipidemia treatments can further complicate lipid management. For example, tolerance to statins—the first-line treatment for dyslipidemia—varies significantly between Asian subgroups. Recent pharmacogenetic evidence has shown that Filipino individuals—and to a lesser extent, Hmong individuals—experience higher systemic exposure to rosuvastatin, which may be attributed to a higher prevalence of the Q141K variant of the *ABCG2* gene (46% in Filipinos, 36% in Hmong) [80]. While statins are considered safe, this higher systemic exposure to rosuvastatin seen in SEA subgroups predisposes them to an elevated risk of statin-associated musculoskeletal symptoms [81, 82]. The reduced tolerance seen in certain SEA populations may contribute to lower treatment adherence, particularly since adverse drug reactions have been widely documented as a leading cause of statin discontinuation [81–84].

Statins are foundational for ASCVD prevention and, while high-intensity therapy shows a small positive HS relationship, the benefits generally outweigh risks [85]. However, because several SEA subgroups have higher systemic rosuvastatin exposure and higher HS rates, clinicians should be more nuanced about initiating or intensifying statin therapy for SEAs [39, 41]. When combined with a heightened baseline susceptibility to intracerebral hemorrhage, aggressive lipid-lowering regimens may compound vulnerability and inadvertently increase cerebrovascular risk in these populations. While the U.S. Food and Drug Administration currently recommends initiating lower rosuvastatin doses for individuals of Asian descent, such guidance remains overly broad and fails to capture the nuances between distinct Asian subgroups [86].

The complex interplay of genetic, sociocultural, and structural determinants underlying dyslipidemia as a major contributor to stroke risk among SEA subgroups underscores the need to shift from generalized CVD prevention in Asian Americans toward tailored, evidence-based interventions. Integrating precision medicine with culturally informed care is essential to addressing subgroup-specific disparities that have long been obscured by data aggregation and underrepresentation.

## Limitations

The greatest limitation of this review is the lack of disaggregated literature on many SEA subgroups, including Thai, Cambodian, Hmong, Laotian, and Burmese populations. Asian Americans have been historically understudied in health research for over 25 years, with most studies focusing on East Asian groups such as Chinese, Japanese, and Korean Americans [87, 88]. Most epidemiologic research to date on CVD risk among SEAs has focused largely on Filipino and Vietnamese Americans—the two largest subgroups in the U.S. population. However, much of this research is based on regional or state-level data, rather than nationally representative samples, as only seven states required disaggregated subgroup reporting prior to 2003 [89, 90]. Moreover, we were unable to identify a robust study reporting the prevalence of high LDL-C, high triglycerides, and low HDL-C in an aggregated Asian American category to serve as a comparison for the subgroup disparities observed in the 2008–2011 cross-sectional lipid profile study [33].

While generalizing findings from Filipino and Vietnamese Americans to other SEA subgroups is problematic—especially given past misinterpretations that assumed all Asian populations share similar health profiles—there is clear evidence of subgroup-specific differences that underscore the urgent need for further research on underrepresented SEA communities [90].

## Conclusion

The high prevalence of dyslipidemia among SEA American subgroups plays a critical role in their elevated risk of CVAs and stroke. This review underscores the multifaceted nature of cardiovascular disease in these communities, shaped by a combination of genetic differences in treatment response, sociocultural influences, structural inequities, and longstanding underrepresentation in health research. While data remain limited for many subgroups, especially beyond Filipino and Vietnamese Americans, the available evidence highlights the importance of improving data disaggregation to reflect the full diversity within the “Southeast Asian” category. Understanding the link between dyslipidemia and stroke through a subgroup-specific lens is essential for improving clinical screening, treatment adherence, and prevention strategies. Ultimately, advancing culturally responsive and precision-based care is a vital step toward reducing cardiovascular disparities and promoting health equity among SEA American populations.

## Key References

- Shah NS, Xi K, Kapphahn KI, Srinivasan M, Au T, Sathye V, et al. Cardiovascular and Cerebrovascular Disease Mortality in Asian American Subgroups. Circ Cardiovasc Qual Outcomes. 2022;15:e008651.
  - *○ There is marked heterogeneity in cardiovascular and cerebrovascular disease mortality among Asian American subgroups. Aggregating Asian American subgroups conceals important differences: e.g.*,* Asian Indian and Filipino individuals in this study had worse or non-improving trends for some outcomes.*
- Vo V, Lopez G, Malay S, Roman YM. Cardiovascular Risk Factors Among Asian Americans: Perspectives on the Role of Acculturation in Cardiovascular Diseases Health Disparities. J Immigr Minor Health. 2024;26:409–20.
  - *○ Although many Asian Americans have relatively favorable socioeconomic indicators*,* they nonetheless may carry substantial burdens of traditional cardiovascular risk factors*,* especially among certain subgroups (e.g.*,* Southeast Asian immigrants)*.
- Nieh H-EV, Roman YM. Major Allele Frequencies in CYP2C9 and CYP2C19 in Asian and European Populations: A Case Study to Disaggregate Data Among Large Racial Categories. J Pers Med. 2025;15:274.
  - *○ This paper shows how aggregating genetically diverse populations under one label masks real biological differences that are clinically meaningful. For example*, saying “Asians have higher CYP2C19 *2 frequency” hides the fact that the allele frequency may be 20% in Japanese,* 35% in Chinese*,* and 50% in Vietnamese*,* which can drastically affect drug response rates in those groups.*
- Bang SH, Huang Y-C, Kuo H-J, Cho ES, García AA. Health status and Healthcare Access of Southeast Asian refugees in the United States: An integrative review. Public Health Nurs. 2023;40:324–37.
  - *○ Refugee populations*,* especially Southeast Asian refugee subgroups*,* are often under-studied in U.S. health research. This review integrates disparate studies to provide a synthesized view of their health status and access issues.*

## Data Availability

No datasets were generated or analysed during the current study.
